# Level of adherence to diet and physical activity among menopausal women and influencing factors in Jordan: a descriptive cross-sectional study

**DOI:** 10.3389/fpubh.2024.1333102

**Published:** 2024-02-02

**Authors:** Rafi Alnjadat, Eshraq Al Momani, Mohammad Etoom, Falastine Hamdan, Salwa Abu ALrub

**Affiliations:** Department of Applied Health Sciences, Al-Balqa Applied University, Al-Salt, Jordan

**Keywords:** diet, exercise, menopause, physical activity, women

## Abstract

**Background:**

During menopause, a woman’s lifestyle may change significantly, which will have implications on her quality of life. Women will experience menopause for more than a third of their life; therefore, maintaining good health during this period is crucial. A healthy diet and physical activity can help women maintain their health during menopause. Hence, assessing adherence to a healthy diet and physical activity among menopausal women is important.

**Purpose:**

This study aims to assess the degree of adherence to a healthy diet and physical activity of menopausal women in Jordan and determine the most influential factors.

**Methods:**

A descriptive cross-sectional design was used in this study, and an online self-administered questionnaire was sent to 299 menopausal women selected through simple random sampling. A valid and reliable questionnaire was used to assess the menopausal women’s adherence to a healthy diet and physical activity. The questionnaire consisted of 14 items covering two domains: 12 questions for the diet domain and two questions for the physical activity domain. Descriptive statistics were obtained, mean weight and body mass index (BMI) were calculated, and stepwise regression was conducted for the data analysis.

**Results:**

The overall degree of adherence to a healthy diet and physical activity of the women was moderate (49.25, *SD* = 7.17). Most of the participants reported eating refined food items once a month or less (*n* = 188, 62.9%) and not exercising weekly (*n* = 119, 39.8%), and only a few reported eating refined food items at least once a day (*n* = 5, 1.7%) and exercising 5–6 times a week (*n* = 15, 5%). The regression analysis showed that age (*B* = 0.145, *p* = 0.014), having two children (*B* = 0.123, *p* = 0.034) and completing primary or secondary education (*B* = 0.120, *p* = 0.038) were statistically significant and the strongest predictors of adherence. The predictors accounted for 68% of the variance in adherence to a healthy diet and physical activity (*R^2^* = 0.068, *F* [343.54] = 7.123, *p* = 0.000).

**Conclusion:**

The majority of the middle-aged menopausal women in this study showed moderate adherence to a healthy diet and physical activity. Age, having two children and completing primary or secondary education were associated with degree of adherence to a healthy diet and physical activity. Therefore, healthcare intervention, such as physical activity and dietary control programs, should target women in this age group and stage in life.

## Introduction

Menopause is a natural transitional period that affects around 1.5 million women annually (1). Menopause is defined as the permanent cessation of ovulation and reduction in estrogen and progesterone owing to diminished ovarian activity (2). The key diagnostic criterion for menopause is the absence of menstrual bleeding within a 12-month period (3). In reality, women spend one third of their life in menopause (4).

Menopause typically begins around the age of 40–60 years (5). The international average age of menopause is 51 years (6). Specifically, the average age of menopause is 51.3 years in European countries, 52.5 years in United States, 48.3 years in South American countries and 51.09 years in Asian countries (7). In Jordan, the average age of natural menopause is 48.5 ± 5.0 years (8). However, the age at menopause may be influenced by the age at menarche, the number of pregnancies, menstrual cycle irregularity, the use of hormonal therapy, the body mass index (BMI), physical activity, smoking, alcohol consumption, socioeconomic status and education level (9, 10).

Estrogen can influence endothelial functions, blood vessel tone and heart functions, as well as the lipid profile and inflammatory status (11). During menopause transition, women may experience troublesome symptoms such as sleep disorders, fluctuating moods, anxiety, fatigue, joint pain, sexual dysfunction, and heart palpitations (12). In addition, vaginal dryness, hot flashes and night sweats are the most frequently reported symptoms (10). Furthermore, the menopausal age is associated with various comorbidities and chronic diseases (13), such as obesity, cardiovascular disease, diabetes, chronic pulmonary disease, osteoporosis, mental illness, depression, dementia and premature death (14–16). Menopause was also reported to be a risk factor of metabolic syndrome (2). Therefore, the proper conditioning of menopausal women’s bodies is important to alleviate symptoms and prevent chronic diseases (17). However, several studies found that regular exercise and food control can alleviate menopausal symptoms and enhance quality of life (18), and obesity and lack of physical activity are associated with decreased quality of life (19).

Certain structural changes that occur in the body during menopause cannot be ameliorated; however, behavior, and lifestyle, specifically, nutrition and physical activity, are among the factors that can be corrected, which can reduce the risk of comorbidities and chronic diseases (20). Adequate nutrition, along with lifestyle adjustment, can help women maintain their health during menopause (21). In addition, adherence to a healthy diet can reduce the risk of metabolic syndrome, heart disease, diabetes, and cancer (22). Similarly, adherence to physical activity may minimize the adverse effect of menopausal symptoms and improve mental health (23). Physical activity may also help reduce weight gain caused by menopause and aging, risk of heart disease and other physical and psychological symptoms, such as body discomfort, exhaustion, poor sleep, and depression (11, 12).

Conversely, insufficient diet and poor physical activity can lead to weight gain and obesity (24), which can increase risk of metabolic diseases and metabolic syndrome and decrease quality of life (6, 19). Therefore, women must maintain a healthy lifestyle, including engaging in exercise; eating low-calorie and low-sodium foods, food that contains appropriate amounts of calcium and vitamin D and fruits, vegetables and fish; and avoiding smoking (25).

Studies have yet to examine factors that can affect adherence to a healthy diet and physical activity among menopausal women in Jordan. Therefore, this study aims to:

- Assess the degree of adherence to a healthy diet and physical activity of menopausal women in Jordan.
- Determine the most influential factors affecting degree of adherence to a healthy diet and physical activity of menopausal women in Jordan.

## Methods

### Design and setting

A descriptive cross-sectional design using an online self-administrated questionnaire was employed in this study to assess the degree of adherence to a healthy diet and physical activity of menopausal women and determine the most influential factors. A sample of 299 menopausal women between the ages of 40 and 60 years were recruited from the northern region. The age range is compatible with the natural age of menopause of women in Jordan, regardless of their marital status, education level, income, and health insurance availability.

### Participants

Probability random sampling through an online questionnaire was conducted to select the participants. The inclusion criteria were as follows: women who were able and willing to participate in the study, her ages are above 40 and less than 60, women who had their last menstrual period 12 months ago and women who could read Arabic. Women with a mental illness, who took medications affect mentality, who experienced artificial menopause owing to radiation, a hysterectomy or an oophorectomy, following a specialized diet were excluded from the research.

The sample size was calculated using a power analysis software based on the medium effect size, the 0.05 significance level and a power level of 0.8 and multiple regression for 15 predictors. Therefore, the sample size should be at least 139 participants (26). The oversampling was intended to overcome attrition or the problem of incomplete responses. Hence, the sample size was set to 167 women.

Google Forms was used for the data collection, and Excel was employed for the data extraction and recording. The participants were recruited via social media and email, that is, the link to the questionnaire was sent to the participants via social media, such as Facebook and WhatsApp. The questionnaire required around 10 min to answer. However, to minimize the risk of response bias, the questionnaires were filled out electronically by participants in the study using a specified tablet.

### Ethical considerations

Approval to collect data was obtained from the Institution Review Board of the Al-Balqa Applied University (number). An online consent form was obtained from each of the participants. The researchers explained the purpose of the study and the expected results and informed the participants that their participation was voluntary, and they had the right to refuse or withdraw from the study at any time without explanation and penalty. Furthermore, the researchers assured the participants that their responses would be confidential.

### Measurement tool

The first part of the instrument collected the participants’ demographic data [i.e., age, education level, employment status, place of residence, governorate, marital status, number of births, economic status, chronic disease, whether they smoked, what they smoked, whether they had undergone hormonal therapy, height, weight, and body mass index (BMI)].

The second part of the instrument, which was developed by Dubasi et al. (27), assessed the women’s adherence to a healthy diet and exercise. The questionnaire consisted of 14 items covering two domains: 12 questions for the diet domain and two questions for the physical activity domain (27).

The responses were measured on a five-point Likert scale (1–5), and the participants with healthy dietary and physical activity habits received a high score, whereas those with poor dietary and physical activity habits received a low score. The score for each question was added to determine the final score (27).

In this study, the scoring system used intervals based on quartiles to determine the degree of adherence to a healthy diet and exercise. A total mean score percentage ranging from 0 to 33.99% indicated poor adherence, a score percentage ranging from 34 to 66.99% indicated moderate adherence and a score percentage of more than 66% indicated satisfactory adherence.

The questionnaire was a valid and reliable tool for assessing adherence to a healthy diet and exercise, the exploratory factor analysis result explained 69.07% of the variance and Cronbach’s alpha was 0.94, which indicated an acceptable internal consistency (27). Furthermore, two qualified translators were recruited to translate the tool into Arabic, and two other qualified translators were recruited to back translated to English and approve the content and format.

A pilot study was conducted to check the readability and psychometric properties of the translated questionnaire. The pilot study involved 11 participants (their data were excluded from the final analysis). The Cronbach’s alpha of the questionnaire was above 0.64.

### Data analysis

Statistical Package for Social Sciences (SPSS; version 26) for Windows was used for the data analysis. Descriptive statistics (mean, standard deviation, and frequency) were used to describe and review the demographic data based on the level of the measurements. Inferential statistics through simple and general stepwise regression were used to identify the factors that may affect adherence to physical activity and a healthy diet among the menopausal women.

## Results

### Participants demographic characteristics

The questionnaire was distributed to 311 menopausal women, 299 of whom completed and returned the questionnaire; thus, the response rate was 96.1%. The mean age of the participants was 44.90 years (*SD* = 7.71), and most of the participants held a BSC degree and a postgraduate degree (*n* = 153, 51.2).

The majority of the participants was a housewife (*n* = 155, 51.8%), was married (*n* = 247, 82.6), lived in town (*n* = 202, 67.6), was from the Irbid Governorate (*n* = 227, 75.9), had three or more births (*n* = 222, 74.2), was a nonsmoker (*n* = 257, 86) and had never undergone hormonal therapy (*n* = 235, 84.6).

Most of the participants in this study were unemployed and expressed dissatisfaction with their monthly income, which ranged from JOD 400 to JOD 800. In addition, the majority of the study participants did not report any chronic disease (*n* = 196, 65.6). The height of the participants was *M* = 1.61 (*SD* = 0.075), their weight was *M* = 75.2 (*SD* = 14.6) and their body mass index (BMI) was *M* = 28.9 (*SD* = 5.74). Table 1 presents the detailed demographic characteristics of the participants.

**Table 1:** Participants demographic characteristics.

Variable	Frequency (*F*)	Percentage (%)	Mean	*SD*
**Age**			44.90	7.71
Educational level				
- Illiterate	6	2		
- Primary or secondary	79	26.4		
- Diploma	61	20.4		
- BSC and postgraduate	153	51.2		
Employment status				
- Employee	138	46.2		
- Housewife	155	51.8		
- Other	6	2		
Place of residence				
- Village	97	32.4		
- Town	202	67.6		
Governorate				
- Irbid	227	75.9		
- Ajlun	40	13.4		
- Jerash	26	8.7		
- Al Mafraq	6	2		
Marital status				
- Married	247	82.6		
- Single	14	4.7		
- Widow	17	5.7		
- Divorced	21	7		
Number of births				
- Nulliparous	26	8.7		
- One	22	7.4		
- Two	29	9.7		
- Three or more	222	74.2		
Economic status				
- Less than 400 JD	110	36.8		
- Between 400 and 800 JD	110	36.8		
- More than 800 JD	79	26.4		
Chronic disease				
- Yes	103	34.4		
- No	196	65.6		
Smoking				
- Yes	42	14		
- No	257	86		
Smoking type				
- Cigarettes	21	7.0		
- Bubbly	19	6.4		
- Electronic cigarettes	2	0.7		
Hormonal thereby				
- Yes, now	30	10		
- Yes, previously	16	5.4		
- No	235	84.6		
Height (M)			1.61	0.075
Weight (Kg)			75.22	14.61
Body mass index			28.96	5.747

*N* = 299.

### Adherence to diet and exercise

The overall total mean score of the menopausal women for adherence to a healthy diet and physical activity was 49.25 (*SD* = 7.17). Specifically, the observed mean score for adherence to a healthy diet was 41.3 (*SD* = 7.21), and the observed mean score for adherence to exercise was 7.86 (*SD* = 2.19; Table 2).

**Table 2:** Level of adherence to diet and exercise.

	Number of items	Actual total score	Mean for observed total score	*SD*
Diet	12	12–60	41.3	7.21
Exercise	2	2–10	7.86	2.19
Total	14	14–70	49.25	7.17

*N* = 299.

Regarding the adherence to a healthy diet and exercise questionnaire items, more than half of the participants (188, 62.9%) reported eating refined food items once a month or less, 174 (58.2%) reported dining out less than once a month, 167 (55.9%) reported eating three meals a day and 160 (53.5%) reported eating ghee, butter, cream and mayonnaise once a month or less (Table 3).

**Table 3:** Adherence to diet and exercise in different items.

Item	*F (%)*
1.How often do you eat meals in a day (including tea, coffee, fruits, salads, snacks)?	
>6 times	14 (4)
6 times	12 (4.7)
5 times	41 (13.7)
4 times	65 (21.7)
3 times	167 (55.9)
2.How often do you drink sweetened beverages like soft drinks, juices, etc.?	
At least once daily	80 (26.8)
3–6 times a week	17 (5.7)
1–2 times a week	70 (23.4)
2–3 times a month	44 (14.7)
Once a month or less	88 (29.4)
3.How often do you eat sweets such as Laddu, Barfi, Jalebi, Kulfi, Chocolate, Halwa, Rice pudding, etc.?	
At least once daily	113 (37.8)
3–6 times a week	41 (13.7)
1–2 times a week	69 (23.1)
2–3 times a month	34 (11.4)
Once a month or less	42 (14)
4.How often do you eat fried foods such as Puri, Parathas, Kachori, Tikki, Bhature, Pakoras, Samosas etc.?	
At least once daily	16 (5.4)
3–6 times a week	36 (13)
1–2 times a week	99 (33.1)
2–3 times a month	58 (19.4)
Once a month or less	87 (29.1)
5.How often do you eat high salty snacks such as Namkeen, Bhujia, Pickles, Chutney, Papad etc.?	
At least once daily	26 (8.7)
3–6 times a week	30 (10)
1–2 times a week	84 (28.1)
2–3 times a month	62 (20.7)
Once a month or less.	97 (32.4)
6.How often do you consume sugar and honey in tea, coffee, curd, lassi, etc.?	
At least once daily	96 (32.1)
3–6 times a week	28 (9.4)
1–2 times a week	42 (14)
2–3 times a month	29 (9.7)
Once a month or less.	104 (34.8)
7.How often do you eat fruit and salad?	
Every time in the main diet	42 (14)
At least once a day	118 (39.5)
3–4 times a week	85 (28.4)
1 time a week	36 (12)
Less than once a week	18 (6)
8.How often do you eat sprouted pulses and green vegetables?	
Every time in the main diet	29 (9.7)
At least once a day	43 (14.4)
3–4 times a week	93 (31.1)
1 time a week	94 (31.4)
Less than once a week	40 (13.4)
9.How often do you eat saturated fat like mutton fat, egg yolks, etc.?	
At least once daily	57 (19.1)
3–6 times a week	72 (24.1)
1–2 times a week	101 (33.8)
2–3 times a month	30 (10)
Once a month or less	39 (13)
10.How often do you eat refined food items like burgers, pizza, etc.?	
At least once daily	5 (1.7)
3–6 times a week	7 (2.3)
1–2 times a week	33 (11)
2–3 times a month	66 (22.1)
Once a month or less	188 (62.9)
11.How often do you eat ghee, butter, cream, mayonnaise, etc.?	
At least once daily	11 (3.7)
3–6 times a week	11 (3.7)
1–2 times a week	49 (16.4)
2–3 times a month	68 (22.7)
Once a month or less	160 (53.5)
12.How often do you eat out of the house (such as wedding, party, family function etc.)?	
More than 3 times a week	8 (2.7)
More than once a week	20 (6.7)
2 times in a month	46 (15.4)
1 time in a month	51 (17.1)
Less than 1 time in a month	174 (58.2)
13.How many days do you exercise in a week?	
Daily	23 (7.7)
5–6 times a week	15 (5)
3–4 times a week	38 (12.7)
1–2 times a week	104 (34.8)
Never	119 (39.8)
14.How much time do you exercise for each session?	
>40 min	20 (6.7)
30–40 min	39 (13)
20–30 min	31 (10.4)
20–10 min	63 (21.1)
<10 min	146 (48.8)

*N* = 299.

Meanwhile, a few of the participants (30; 10%) reported eating salty snacks 3–6 times a week; 30 (10%) reported eating saturated fats 2–3 times a month; 29 (9.7%) reported eating sprouted pulses and green vegetables in every meal as their main diet; 28 (9.4%) reported consuming sugar in their coffee and tea 3–6 times a week; 18 (6%) reported eating fruits and a salad less than once a week; 17 (5.7%) reported drinking sweetened beverages 3–6 times a week; 16 (5.4%) reported eating fried food at least once a day; 11 (3.7%) reported eating ghee, butter, cream, and mayonnaise at least once a day and 3–6 times a week; 8 (2.7%) reported dining out more than three times a week; and 5 (1.7%) reported eating refined food items at least once a day.

In terms of exercise, most of the participants (119; 39.8%) reported not exercising weekly, and 146 (48.8%) reported participating in an exercise session that lasted less than 10 min. Few participants reported exercising 5–6 times a week (15; 5%), and 20 (6.7%) reported participating in an exercise session that lasted more than 40 min.

### Factors predicting adherence to diet and physical activity

The results of the simple linear regression analysis revealed that age (*B* = 0.206, *p* = 0.000), completing primary or secondary education (*B* = 0.146, *p* = 0.010), having BSC and postgraduate degrees (*B* = −0.148, *p* = 0.009), having two children (*B* = −0.167, *p* = 0.003), smoking electronic cigarettes (*B* = −0.119, *p* = 0.036) and body mass index (BMI) (*B* = 0.136, *p* = 0.019) were significant predictors of adherence to a healthy diet and physical activity (Table 4). All the predictors with a *p < 0.25* value were entered into another model for the stepwise regression analysis.

**Table 4:** Predictors of adherence to diet and physical activity using simple linear regression.

		For predictor				For model			
	Predictors	Coefficients Beta	*t*	*p*	*F*	*p*	*R*	*R^2^*	Adjusted *R^2^*
	Age	0.206	3.68	0.000**	13.56	0.000	0.206	0.042	0.039
	Educational level								
	- Illiterate vs. primary or secondary	0.146	2.58	0.010**	6.68	0.010	0.146	0.021	0.018
	- Illiterate vs. diploma	0.019	0.329	0.742	0.108	0.742	0.019	0.000	−0.003
	- Illiterate vs. BSC and postgraduate	−0.148	−2.635	0.009**	6.943	0.009	0.148	0.022	0.019
Model 1	Employment status								
	- Employee vs. housewife	0.065	1.148	0.252	1.317	0.252	0.065	0.004	0.001
	- Employee vs. other	0.028	0.498	0.619	0.248	0.619	0.028	0.001	−0.002
	Place of residence								
	- Village = 0	0.080	1.405	0.161	1.973	0.161	0.080	0.006	0.003
	- Town = 1								
	Governorate								
	- Irbid vs. Ajlun	−0.036	−0.623	0.530	0.395	0.036	0.001	−0.002	−0.530
	- Irbid vs. Jerash	0.022	0.381	0.703	0.145	0.703	0.022	0.000	−0.003
	- Irbid vs. Al-Mafraq	−0.032	−0.556	0.578	0.310	0.578	0.032	0.001	−0.002
	Marital status								
	- Married vs. single	0.013	0.224	0.823	0.050	0.823	0.013	0.000	−0.003
	- Married vs. widow	0.092	1.615	0.107	2.609	0.107	0.092	0.008	0.005
	- Married vs. divorced	−0.044	−0.776	0.438	0.602	0.438	0.044	0.002	−0.001
	Number of births								
	- Nulliparous vs. one	0.031	0.546	0.585	0.298	0.585	0.031	0.001	−0.002
	- Nulliparous vs. two	−0.167	−2.976	0.003**	8.855	0.003	0.167	0.028	0.025
	- Nulliparous vs. three or more	0.088	1.556	0.121	2.421	0.121	0.088	0.008	0.005
Model 1	Economic status								
	- Less than 400 vs. between 400–800	−0.070	−1.227	0.221	1.506	0.221	0.070	0.005	0.002
	- Less than 400 vs. more than 800	0.023	0.407	0.684	0.166	0.684	0.023	0.001	−0.003
	Chronic disease								
	- Yes = 0	−0.071	−1.241	0.216	1.539	0.216	0.071	0.005	0.002
	- No = 1								
	Smoking								
	- Yes = 0	0.036	0.641	0.522	0.411	0.522	0.036	0.001	−0.002
	- No = 1								
	Smoking type								
	- Cigarette vs. bubbly	−0.055	−0.959	0.338	0.920	0.338	0.055	0.003	0.000
	- Cigarette vs. electronic cigarette	−0.119	−2.103	0.036**	4.424	0.036	0.119	0.014	0.011
	Hormonal therapy								
	- Yes, now vs. yes previously	−0.002	−0.031	0.975	0.001	0.975	0.002	0.000	−0.003
	- Yes, now vs. no	−0.069	−1.220	0.223	1.488	0.223	0.069	0.005	0.002
	Body mass index (BMI)	0.136	2.364	0.019**	5.589	0.019	0.136	0.018	0.015

*p* < 0.05 (two-tailed). Significant value of less than 0.05.

The results of the general stepwise regression revealed that age (*B* = 0.145, *p* = 0.014), having two children (*B* = 0.123, *p* = 0.034) and completing primary or secondary education (*B* = 0.120, *p* = 0.038) were statistically significant and the strongest predictors of adherence. The predictors accounted for 68% of the variance in adherence to a healthy diet and physical activity (*R^2^* = 0.068, *F* [343.54] = 7.123, *p* = 0.000; Table 5).

**Table 5:** Predictors of adherence to diet and physical activity using stepwise linear regression.

	Predictors	Coefficients Beta	(95%CI)	*t*	*p*	*F*	*p*	*R*	*R^2^*	Adjusted *R^2^*
	Age	0.145	(0.025, 216)	2.749	0.014**					
Model 2	Number of births									
	- Nulliparous vs. two	0.123	(−5.709, −0.218)	−2.124	0.034**	7.123	0.000	0.260	0.068	0.058
	Educational level									
	- Illiterate vs. primary or secondary	0.120	(0.115, 3.850)	2.089	0.038**					

Model 2: *F* (343.54) = 7.123; *df* = (3); *R*^2^ = 0.068; Adjusted *R*^2^ = 0.058, *p* = 0.000 (two-tailed). Significant value of less than 0.05.

Result from general stepwise regression revealed that age (*B* = 0.145, *p* = 0.014), had two children (*B* = 0.123, *p* = 0.034), had a primary or secondary education (*B* = 0.120, *p* = 0.038), are statistically significant and strongest and predictors. These predictors account for 68% variance in adherence to diet and physical activity (*R^2^* = 0.068, *F* (343.54) = 7.123, *p* = 0.000; Table 5).

## Discussion

This study aims to assess the degree of adherence to a healthy diet and physical activity of menopausal women in Jordan and determine the most influential factors. Healthy diet and exercise can play a vital role in the health of women during menopause. Adherence to a healthy diet and exercise can minimize the risk of chronic diseases and comorbidities and play a crucial role in managing lifestyle-related diseases (27).

In this study, the women’s degree of adherence to a healthy diet and exercise was moderate, this could be related to that most of study participants were married and housewife’s, which means they spend most of their time doing housework and taking care of family. In addition, this may be related to their different health status, ages, education levels and levels of awareness of the benefits and advantages of a healthy diet and exercise. Physical activity was influenced by the women’s age, place of residence, education level, occupation, marital status, body mass index (BMI), parity and socioeconomic status (4, 28). This result is consistent with that of previous studies that observed moderate physical activity among the participants (6, 9, 29). By contrast, other studies observed sedentary physical activity and poor diet among their study participants (30–33).

A healthy diet consists of food with fiber, water, vitamins, minerals, proteins, carbohydrates, and fats (29). A healthy diet can reduce the risk of diseases and enhance quality of life (34). Moreover, adequate nutritional fulfillment can considerably reduce the negative consequences of menopause (29). The results of the present study revealed that more than half of the participants ate refined food items once a month or less, dined out less than once a month, ate three meals a day and ate ghee, butter, cream, and mayonnaise once a month or less. This result was satisfactory, because insulin resistance is likely to develop in individuals who consume large amounts of refined carbohydrates (14). Consumption of refined grains, foods high in saturated fats, desserts, and beverages sweetened with sugar can result in severe menopausal symptoms (35). Thus, a possible explanation for the result is that most of the participants were housewives and highly educated. This result is consistent with that of a previous study that found that most of the participants did not skip meals and followed a meal pattern (33, 36). Furthermore, a previous study determined that the intake of mayonnaise and liquid oils of the participants was high (37). By contrast, another study observed that high-calorie foods such as fats, bread, cereals, sweets, meat and oil were consumed in excess amounts by the participants (9), and Tasleem et al. (38) reported that the most consumed food group in their study was dairy.

Meanwhile, few participants reported eating salty snacks 3–6 times a week, eating saturated fats 2–3 times a month, eating sprouted pulses and green vegetables in every meal as their main diet, consuming sugar in their coffee and tea 3–6 times a week, eating fruits and a salad less than once a week, drinking sweetened beverages 3–6 times a week and eating fried food at least once a day. This result indicated that the participants had poor dietary habits. Low fruit and vegetables intake can affect bone density and thus increase the risk of osteoporosis. Fruits and vegetables include antioxidants that can mitigate the negative effects of reactive oxygen species on the quantity and quality of ovarian follicles; thus, they can lengthen the reproductive lifespan (32). In addition, low-intensity menopausal symptoms are associated with high consumption of healthy grains and vegetables (34). To avoid heart and metabolic problems, the appropriate consumption of calcium and foods containing calcium, such as dairy products, fruits, and vegetables, should be emphasized (39). Thus, early nutritional instruction is required, because a woman’s bad eating habits may continue into the menopausal stage (34).

This result is consistent with that of a previous study that found that the intake of fruits and vegetables was low among the participants (9, 32, 34, 36, 38, 40). Conversely, another study reported the lowest intake for proteins, carbohydrates, fats, calcium, magnesium, phosphorus and iron among its participants (33).

High and low levels of physical activity can impact estrogen production. Therefore, women should be encouraged to consider their degree of physical activity (29). The results of the present study revealed that most of the participants did not exercise weekly, and each exercise session lasted less than 10 min. Meanwhile, few participants reported exercising 5–6 times a week, with each exercise session lasting more than 40 min. This result may be explained by the overweight state and age of the participants. The skeletal muscle fatty acid intake system and β oxidation pathways are activated during exercise, which can help enhance energy expenditure and reduce body fat (17). The absorption and use of glucose by skeletal muscles can be boosted by exercise (17). Thus, exercise is important for weight loss and can minimize the risk of comorbidities. This result is consistent with that of a previous study that showed that the majority of the female participants did not engage in physical activity (14, 32). By contrast, Lewandowska et al. (19) reported that the majority of the participants in their study had an adequate level of exercise, that is, more than 600 metabolic equivalents min/week.

According to the study results, a relationship existed between age, completing primary or secondary education, having BSC and postgraduate degrees, having two children, smoking electronic cigarettes, the body mass index (BMI), and adherence to a healthy diet and physical activity during menopause.

In this study, a positive relationship was observed between age and adherence to a healthy diet and physical activity, which may be related to the women’s understanding of the importance of adherence owing to their wisdom and life experiences, which may increase their awareness of the benefits of long-term adherence. In addition, having a healthy lifestyle and well-being are prioritized with age. The mean age of the study participants was 44.90 years, which is consistent with that in a previous study in Jordan that recorded the mean age at 48.5 ± 5.0 years (8). Al-Smadi (6) reported that women between the ages of 45 and 50 years accounted for the highest proportion of the total population of menopausal women in Jordan. This result is similar to that of Ranasinghe et al. (32), who recorded the mean age as 49.9 ± 3.9 years, and that of Tiwari et al. (33), who recorded the mean age as 48.58 ± 3.38 years. However, in the study of Assaf et al. (1), the mean age of the Jordanian female participants was 50.5 ± 4.8 years. Meanwhile, Chen et al. (41), Dunneram et al. (14), Moradi et al. (42), Nournezhad et al. (9), and Ozcan (25) found that the mean age of the participants in their study was above 50 years. Conversely, other studies reported a mean age of less than 45 years (43).

Women with a high education level may be highly knowledgeable about health-related topics and healthy lifestyles (25). Thus, women’s health promotion and preventative behaviors were found to be positively affected by their education level (44). In addition, highly educated women may be conscious of menopausal symptoms and the psychological and physical changes that occur during menopause (45). In the present study, most of the participants had BSC and postgraduate degrees, but a negative relationship was observed between having BSC and postgraduate degrees and adherence to a healthy diet and physical activity, and a positive relationship was observed between completing primary or secondary education and adherence to a healthy diet and physical activity. This result contradicts the belief that a woman with a high education level will demonstrate high adherence, which may be related to the different sample sizes between the two categories. Moreover, a high percentage of the study participants were employees, had three or more births and had low or middle-level income. Such factors may limit the women’s time and money for adherence to a healthy diet and physical activity. This result is similar to that of a previous study in Jordan that reported that most of the female participants had a BSC degree (1, 8). Previous studies also found that most of the female participants completed primary education or had an education level that was below high school (9, 19, 25, 32, 33, 41, 43).

In this study, most of the participants were married, were housewives and had more than three children, which may explain their lack of physical activity and exercise. In addition, a positive relationship was observed between having two children and adherence to a healthy diet and physical activity. This result contradicts previous findings that revealed that having children may prevent women from exercising regularly and having a healthy lifestyle (41). Jordanian women are responsible for various aspects of their family life, including caring for their grandchildren and their aging parents and in-laws (1). The result of the present study is consistent with that of a previous study that found that most of the menopausal participants were married (1, 8, 32, 33, 41, 43). Another study in Jordan observed that most of the menopausal participants were housewives and had children (8). More et al. (36), Nournezhad et al. (9), Ozcan (25), Ranasinghe et al. (32), and Tasleem et al. (38) also found that most of the menopausal women in their study were housewives. However, Assaf et al. (1) reported that most women in Jordan were employed and had five or more children. Other studies reported that most menopausal women were retired and had two children (19).

Employment can increase a woman’s total family income and empowerment (1). Women’s satisfaction with their income is reflected in their use of health services and compliance with preventive health measures (46). In the present study, most of the participants had a monthly income of less than JOD 800. However, Bustami et al. (8) found that most menopausal women in Jordan had a monthly income of less than JOD 500. Ranasinghe et al. (32) reported that most of their study participants were from the upper and upper-middle class. However, Assaf et al. (1) found that perceived family income in Jordan was unsatisfactory, and Tiwari et al. (33) observed that the economic status of their research participants was low.

In the present study, most of the participants were nonsmokers and had never undergone hormonal therapy, which are in line with the results of a previous study in Jordan that reported that most of the female participants were nonsmokers and had never undergone hormonal therapy (8). Galfo et al. (43), Lewandowska et al. (19), Nournezhad et al. (9), Ozcan (25), and Ponichter et al. (34) also found that most women are nonsmokers. However, in the present study, a significant negative relationship was observed between electronic cigarette smoking and adherence to a healthy diet and physical activity. This finding may be related to the fact that electronic cigarettes contain nicotine, which is an addictive substance that may affect the brain chemistry and distract women from maintaining their healthy lifestyle habits. Another factor that may cause women to ignore the importance of adherence to a healthy diet and physical activity may be the false claims made by electronic cigarettes, such as optimism, relaxation and stress relief.

The mean body mass index (BMI) of the study participants was 28.96, which meant that they were overweight. This finding could be related to the participants’ lack of regular physical activity, as most reported that they never exercised, and those who exercised reported participating in an exercise session that lasted less than 10 min. However, physical activity can reduce the risk of obesity (41). Thus, interventions to improve physical activity should be suggested to address the increasing prevalence of obesity (32). This result is similar to that of Bustami et al. (8), who found that the majority of Jordanian menopausal women is overweight. Previous studies also reported the overweight state of their female participants (15, 19, 25, 34). Another study reported that 93% of its female participants had a higher-than-normal body mass index (BMI) (9), but other studies reported standard and normal BMIs among their participants (14, 32, 38, 41, 43).

### Strength and limitations

The present study is the first to assess the degree of adherence to a healthy diet and physical activity of menopausal women in Jordan and determine the most influential factors.

The sample size, response rate of the study was sufficient, sampling recruitment was from northern region. Adherence to healthy lifestyle are an effective strategy to delay and prevent the development and progression of menopausal symptoms in Jordan. This study identifies the most confounding factors that might impact the development of healthy intervention program.

Nevertheless, this study has limitations that should be considered when interpreting the findings. Firstly, the adopted cross-sectional quantitative design may not be sufficient to identify all the potential factors that may influence adherence to a healthy diet and physical activity among the menopausal women. Secondly, this study provides a limited representation of the female population of Jordan, focusing on specific regional attributes. Consequently, this could restrict the generalizability of the study’s findings. Thirdly, the use of an online questionnaire may limit the generalizability of the results. In addition, the self-reported weight and height may be underestimated or overestimated. Lastly, other activities that occurred concurrently with the survey may affect the results, and “history” may have influenced how the women responded to the survey questions (47).

### Conclusion

This study increases our understanding about the level of adherence to diet and physical activity among the menopausal women. Also, fills knowledge gap in the literature, and enhance our awareness of the requirements to improve adherence to diet and physical activity. In addition, it sheds light in the factors affecting adherence to diet and physical activities. This study demonstrated a moderate degree of adherence to a healthy diet and physical activity among menopausal women.

Age, having two children and completing primary or secondary education were statistically significant and the strongest predictors of adherence to a healthy diet and physical activity among the menopausal women. Future research should validate the results of this study and investigate other factors that may affect adherence to a healthy diet and physical activity among menopausal women. Also, conducting a future interventional studies to provide menopausal women with information and education related to physical activity and a healthy diet, and increase their awareness regarding the importance of healthy diet and physical activity. Furthermore, Suggestions can be offered to healthcare professionals for developing personalized strategies for lifestyle adjustment, as well as comprehensive interventions that encompass dietary regulation and physical activity for menopausal women. Moreover, mixed-methods and qualitative studies should be conducted to improve understanding of the factors that may predict adherence to a healthy diet and physical activity.

## Data availability statement

The raw data supporting the conclusions of this article will be made available by the authors, without undue reservation.

## Ethics statement

The studies involving humans were approved by the Institution Review Board of the Al-Balqa Applied University which assigned the approval number 5/6/23. The studies were conducted in accordance with the local legislation and institutional requirements. The participants provided their written informed consent to participate in this study.

## Author contributions

RA: Conceptualization, Formal analysis, Visualization, Writing – original draft. EM: Investigation, Methodology, Project administration, Writing – review & editing. ME: Conceptualization, Data curation, Validation, Writing – original draft. FH: Conceptualization, Data curation, Supervision, Validation, Writing – review & editing. SA: Data curation, Formal analysis, Methodology, Writing – review & editing, Investigation.
